# Postpartum Seronegative Autoimmune Hepatitis: Diagnostic Challenges and Clinical Implications

**DOI:** 10.1016/j.gastha.2026.101055

**Published:** 2026-07-09

**Authors:** Aya Fadel, Yanie Oliva, Jose A. Gascon, Zahraa F. Saadoon, Lamia Nasser, Dima Nathir Dawood Sarah, Robert J. Hernandez, Arumugam R. Jayakumar, Hussain Hussain

**Affiliations:** 1 Department of Internal Medicine, HCA Florida Capital Hospital, Tallahassee, Florida; 2 Department of Internal Medicine, HCA Florida Kendall Hospital, Miami, Florida; 3 Department of Obstetrics, Gynecology and Reproductive Sciences, School of Medicine, University of Miami Miller, Miami, Florida

**Keywords:** Autoimmune hepatitis, Liver, AST, ALT, Postpartum

## Abstract

Postpartum autoimmune hepatitis (AIH) is a rare but serious condition triggered by immune rebound after delivery. Diagnosis is typically supported by elevated immunoglobulins and positive autoimmune markers; however, seronegative presentations remain diagnostically challenging. We present the fourth reported worldwide case of seronegative postpartum AIH in a 36-year-old woman who developed jaundice, pruritus, pale stools, and dark urine 4 months after delivery. Laboratory evaluation revealed severe hepatocellular injury with aspartate aminotransferase 1373 U/L, alanine aminotransferase 2137 U/L, and marked hyperbilirubinemia. Viral, toxic, metabolic, and rheumatologic investigations were negative, while autoimmune serologies and immunoglobulin levels remained normal. Imaging demonstrated hepatomegaly without biliary obstruction or vascular abnormalities. Liver biopsy revealed acute-on-chronic autoimmune-mediated hepatitis with necrosis, confirming seronegative postpartum AIH. This case emphasizes seronegative AIH as a critical consideration in postpartum acute hepatitis, with liver biopsy essential for diagnosis and early treatment.

## Introduction

Postpartum autoimmune hepatitis (AIH) is a rare but clinically significant condition characterized by immune reactivation after delivery, often leading to acute or severe hepatic inflammation in women who may have had stable liver function during pregnancy.^1^, ^2^, ^3^ The condition is marked by aberrant innate and adaptive immune responses targeting hepatocytes, resulting in progressive hepatic inflammation and potential liver failure if untreated.^3^^,^^4^ It predominantly affects women of reproductive age, making pregnancy and the postpartum period critical windows for disease expression.^1^ During pregnancy, immunological shifts toward a T helper (Th)2-dominant, tolerance-oriented state often suppress autoimmune activity, which explains why many patients experience remission or stabilization.^4^ Nevertheless, the postpartum period reverses this immune tolerance, triggering heightened immune reactivity and increasing the risk of AIH flare-ups in up to 55% of cases.^4^

Postpartum AIH may present as a new-onset disease or as a flare of previously quiescent AIH.^4^^,^^5^ Positive autoimmune serologies such as anti-smooth muscle antibodies or anti-F-actin antibodies, which are specific markers aiding diagnosis.^4^ While seronegative AIH can only be diagnosed via histopathological findings typically include interface hepatitis, lymphoplasmacytic infiltration, and varying degrees of necrosis.^6^ Clinically, postpartum AIH can range from mild hepatic cytolysis to severe or fulminant presentations, sometimes requiring urgent immunosuppressive therapy. Corticosteroids remain the first-line treatment, often producing rapid biochemical improvement, while azathioprine may be added for long-term control.^7^

Postpartum AIH represents a diagnostic and therapeutic challenge. Early recognition is essential to prevent progression to liver failure and to ensure favorable maternal outcomes. This case disclosed the crucial need for vigilant postpartum monitoring, timely immunological workup, and prompt initiation of therapy to mitigate disease severity and preserve long-term liver function.

## Case Presentation

A 36-year-old woman, gravida 2 para 2, presented to the emergency department 4 months postpartum with a 2-week history of progressive diffuse jaundice associated with generalized pruritus, dark urine, pale stools, and worsening fatigue. She denied abdominal pain, fever, nausea, vomiting, or other gastrointestinal symptoms. The patient denied use of prescription medications, over-the-counter agents, herbal supplements, recreational drugs, or recent exposure to hepatotoxic substances. She also denied alcohol consumption, prior liver disease, recent travel, sick contacts, blood transfusions, high-risk sexual exposure, prior anesthesia exposure, or recent surgical procedures. There was no personal history of autoimmune disease, including thyroid disease, inflammatory arthritis, connective tissue disorders, or previous episodes of hepatitis. Furthermore, expanded infectious workup, including Epstein–Barr virus, cytomegalovirus, herpes simplex virus, and other viral serologies, was negative. Toxicology screening and rheumatologic testing were also negative. Immunoglobulin levels were within normal limits, and autoimmune serologies, including antimitochondrial antibody and anti–smooth muscle antibody, anti-actin antibody, anti-liver-kidney microsomal type 1 antibody, anti-liver cytosol type 1, and anti-mitochondrial antibody, were negative. Ceruloplasmin and alpha-fetoprotein levels were likewise within normal limits. Despite the negative serologic investigation, initial laboratory evaluation demonstrated severe acute hepatocellular injury, with aspartate aminotransferase 1373 U/L (normal <40 U/L), alanine aminotransferase 2137 U/L (normal <60 U/L), alkaline phosphatase 178 U/L (normal <130 U/L), international and normalization ratio was 1.4. Total bilirubin was markedly elevated at 14.7 mg/dL (normal <1.2 mg/dL), with a direct bilirubin level of 7.4 mg/dL.

**Figure 1:**
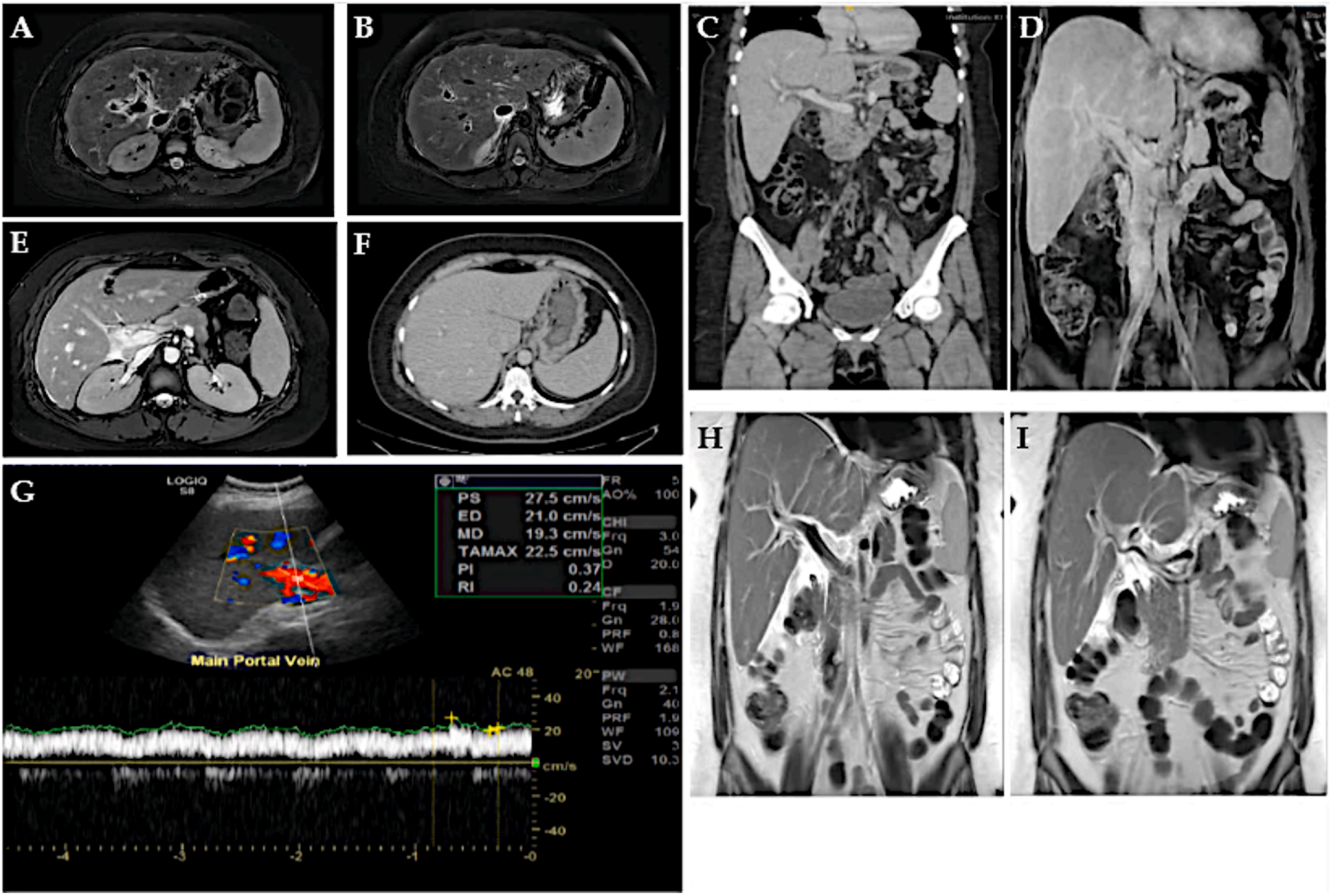
Radiographic findings disclosed mild hepatomegaly on MRI (A, B, D, E) and CT imaging (C and F). Furthermore, magnatic resonance cholangiopancreatography (MRCP) showed a patent biliary tree without evidence of obstruction (H and I), and the doppler ultrasound of portal vein was negative for thrombosis (G). CT, computer tomography; MRI, magnetic resonance imaging.

**Figure 2:**
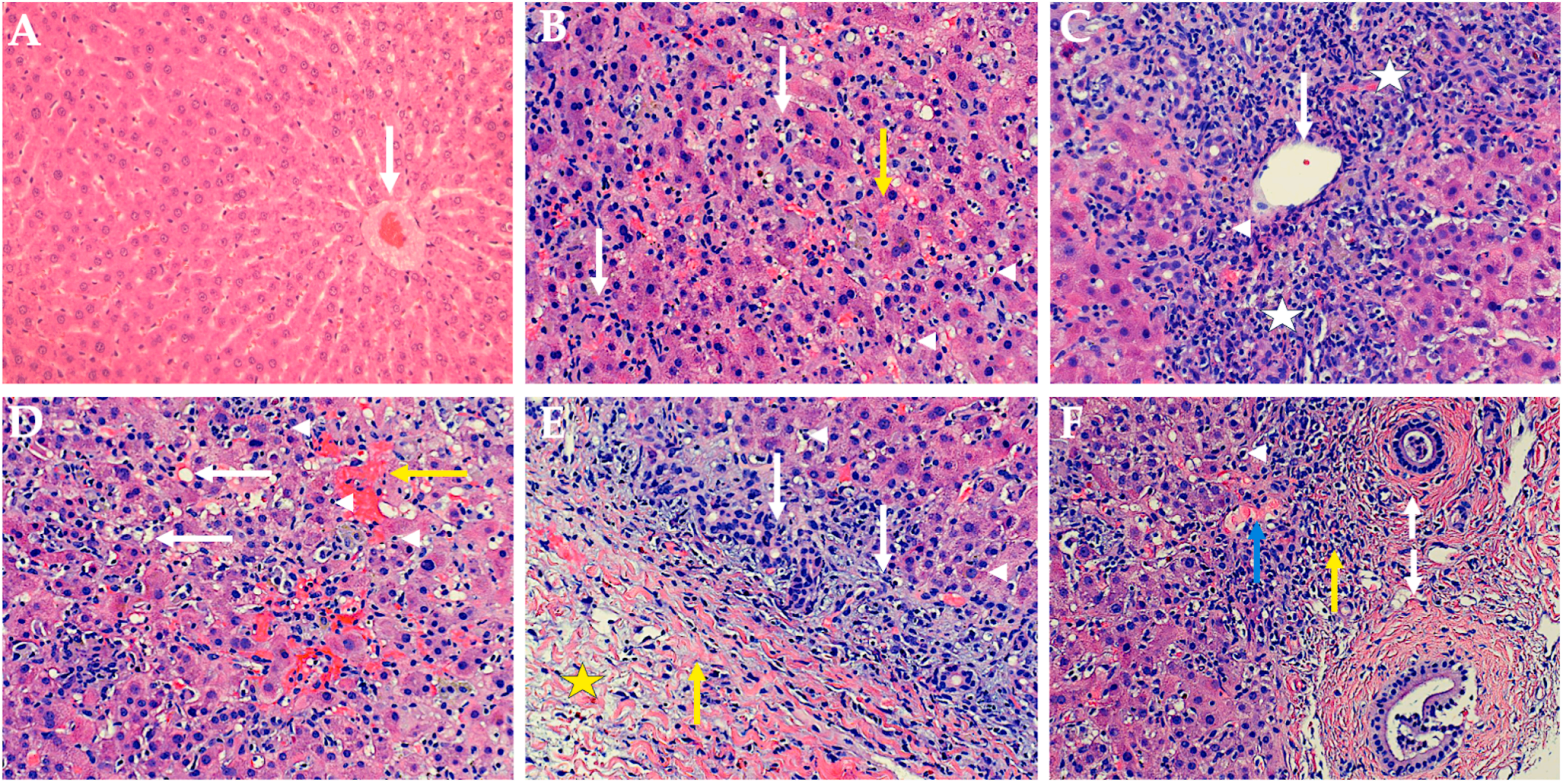
Hematoxylin and eosin (H&E) staining of the liver demonstrated preserved hepatic architecture in Panel (A) without significant inflammatory changes, particularly surrounding the portal vein (white arrow). Panel (B) revealed focal microhemorrhage (yellow arrow) with extensive scattered lymphocytic infiltration (white arrow). Panel (C) demonstrated the portal vein (white arrow) surrounded by prominent inflammatory cell infiltration (white star). Panel (D) showed additional areas of microhemorrhage (yellow arrow) associated with tissue edema and vacuolization (white arrow). Panel (E) illustrated features bridging acute and acute-on-chronic hepatic injury, with inflammatory cell aggregates (white arrows) extending across areas of collagen deposition and fibrosis (yellow arrow and yellow star). Panel (F) demonstrated extensive fibrosis and collagen deposition surrounding vascular structures, accompanied by persistent inflammatory infiltrates and focal microhemorrhage. Panels (B–F) (white arrowheads) demonstrate plasmacytic cellular infiltration within the hepatic tissue.

Imaging studies revealed mild hepatomegaly without biliary obstruction or vascular abnormalities. Magnetic resonance imaging and CT scans showed no focal lesions, and Doppler ultrasound confirmed patent portal venous flow without thrombosis (Figure 1). Given the severity of liver injury and absence of identifiable etiology, a liver biopsy was performed. Histopathology demonstrated acute on chronic hepatitis with necrosis, consistent with autoimmune-mediated hepatic injury in the postpartum setting despite negative serology (Figure 2).

The patient was initiated on corticosteroid therapy along with supportive management. Due to the risk of progression to acute liver failure, gastroenterology recommended transfer to a liver transplant center for advanced care. However, the patient declined the transfer and subsequently left the hospital against medical advice despite extensive counseling regarding the potential risks.

## Discussion

AIH is an uncommon but clinically important entity that emerges in the months following delivery, a period characterized by rapid immunologic rebound after the relative immune tolerance of pregnancy.^6^, ^7^, ^8^ Although AIH typically presents with elevated immunoglobulin G levels and positive autoimmune serologies, commonly anti-smooth muscle antibodies or antinuclear antibody, whereas, some patients exhibit seronegative AIH, a diagnostic challenge that requires careful clinical correlation and histopathologic confirmation.^9^ The present case illustrates this rare phenotype: a 36-year-old postpartum woman with severe acute hepatitis, negative autoimmune markers, and biopsy-proven autoimmune-mediated hepatic injury. The patient’s presentation 4 months postpartum aligns with the well-recognized window during which immune reactivation can precipitate autoimmune disease or trigger flares of previously silent conditions. The abrupt onset of jaundice, pruritus, pale stools, and dark urine, combined with markedly elevated aminotransferases and significant hyperbilirubinemia, is consistent with severe hepatocellular injury. The absence of fever, abdominal pain, or systemic symptoms further supports a noninfectious inflammatory etiology.

Pathophysiology is complex, AIH occurs after delivery, when the maternal immune system rapidly shifts from the immunotolerant state of pregnancy back to a proinflammatory state.^10^^,^^11^ During pregnancy, elevated levels of estrogen, progesterone, cortisol, and regulatory T cells suppress autoreactive immune responses to maintain tolerance to the semi-allogeneic fetus.^11^^,^^12^ Following delivery, this mechanistic of immunosuppression abruptly declines, leading to immune rebound activation characterized by increased Th1 and Th17 activity, reduced regulatory T-cell function, and enhanced autoantibody production.^13^^,^^14^ In certain patients, this immune reconstitution may trigger loss of tolerance to hepatocyte antigens, resulting in lymphoplasmacytic infiltration of the liver, interface hepatitis, and progressive hepatocellular injury.^13^ Elevated inflammatory cytokines, including tumor necrosis factor-α and interferon-γ, further amplify hepatic inflammation and fibrosis.^13^ Some patients may present with seronegative disease, in which conventional autoantibodies are absent despite histologic findings consistent with AIH, suggesting that cell-mediated immune mechanisms may predominate in certain postpartum cases.^6^ Only 3 AIH cases related to seronegativity, particularly in acute or fulminant presentations where antibody titers may be low or transiently absent.^6^^,^^13^ In such cases, liver biopsy becomes the decisive diagnostic tool.^6^ This is the fourth case so far representing AIH in a seronegative state.

Histopathology in this patient demonstrated acute on chronic hepatitis with necrosis, a highly suggestive pattern of autoimmune-mediated injury in the appropriate clinical context.^15^^,^^16^ Interface hepatitis, lymphoplasmacytic infiltration, and hepatocyte necrosis are hallmark features of AIH, even when serologies are negative.^15^^,^^16^ The postpartum timing further strengthens the diagnosis, as immune rebound after delivery is known to precipitate both new-onset and flare-ups of AIH.

This case emphasizes several important clinical aspects. Initially, postpartum hepatitis should prompt consideration of autoimmune etiologies, even when serologic markers are absent. Also, seronegative AIH remains a diagnosis of exclusion, requiring integration of clinical presentation, laboratory data, imaging, and histopathology. Third, early recognition and initiation of corticosteroid therapy are essential to prevent irreversible liver injury. Hence, the case highlights the challenges clinicians face when patients decline recommended care, emphasizing the need for clear communication about risks, close outpatient follow-up, and strategies to support shared decision-making.
